# Impact of Peripheral Refractive Errors in Mobility Performance

**DOI:** 10.1167/iovs.65.6.42

**Published:** 2024-06-28

**Authors:** Clara García-Pedreño, Juan Tabernero, Antonio Benito, Pablo Artal

**Affiliations:** 1 Departamento de Electromagnetismo y Electrónica, Universidad de Murcia, Murcia, Spain; 2 Laboratorio de Óptica, Universidad de Murcia, Murcia, Spain

**Keywords:** peripheral vision, peripheral refraction, mobility, gait

## Abstract

**Purpose:**

The purpose of this study was to investigate the functional effects of peripheral refractive errors on mobility performance through a stair negotiation task.

**Methods:**

Twenty-one young, normal sighted subjects navigated through an obstacle with steps, wearing spectacles that altered only their peripheral refraction. Lenses were used to induce positive defocus (+2 diopters [D] and +4 D), negative defocus (−2 D and −4 D), or astigmatism (+1.75 D and −3.75 D, axis 45 degrees) in the periphery. Feet trajectories were analyzed, and several gait assessment parameters were obtained. Statistical tests were conducted to determine significant performance differences between the lenses. Peripheral refraction in each subject was measured using a scanning Hartmann-Shack wavefront sensor to assess the impact of intrinsic peripheral refraction on the experiment.

**Results:**

Statistically significant differences in performance appeared when peripheral errors were superimposed. Crossing time with respect to plano lenses increased by 6.2%, 7.6%, 19.2%, and 29.6% for the −2 D, +2 D, −4 D, and +4 D lenses, respectively (*P* < 0.05 in the last 3 cases). Subjects exhibited slower walking speeds, increased step count, and adopted precautionary measures. High-power positive defocus lenses had the biggest impact on performance, and differences were observed in distance to steps between induced positive and negative defocus.

**Conclusions:**

In this laboratory-based study without an adaptation period, peripheral refractive errors affected stair negotiation, causing cautious behavior in subjects. Performance differences among types of peripheral defocus may result from magnification effects and intrinsic peripheral refraction. These results highlight the importance of understanding the effects of induced peripheral errors by myopia control and intraocular lenses.

Human vision is optimized for the central (foveal) retina, which has a higher density of photoreceptors and ganglion cells and can therefore perform high-resolution tasks. On the other hand, peripheral vision has much poorer optical quality due to increasing peripheral refractive errors,^1^ and especially the limited density of ganglion cells and photoreceptors.^2^^,^^3^ Despite the limitations of peripheral vision, it is essential for performing tasks, such as saccade planning, track multiple objects at once, object recognition, or gist recognition of a scene.^4^^,^^5^ Overall, good peripheral vision is useful in sports,^6^ driving,^7^ or guiding locomotion.

In the case of gait, vision is necessary for on-line guidance of locomotion,^8^ and peripheral vision plays an essential role in adapting to changes in ground terrain and adjusting foot placement and clearance over obstacles.^9^ Of the entire peripheral visual field, the lower visual field is crucial for safe stair negotiation because it may contain more information about the position of the lower extremities. Studies show that lower visual field information is used to plan kinematic parameters,^10^ and may even be sufficient to guide locomotion when navigating steps.^11^

In recent years, more attention has been paid to peripheral optics. Myopic eyes tend to be relatively hyperopic in the periphery due to the elongated shape of the eye, whereas hyperopic and emmetropic eyes are more myopic in the periphery.^12^^,^^13^ Many optical treatments to slow the progression of myopia rely on the introduction of peripheral myopic defocus to compensate for the intrinsic hyperopic blur and slow axial growth.^14^^,^^15^ On the other hand, intraocular lenses (IOLs) implanted in cataract surgery to replace the crystalline lens, induce peripheral myopia and astigmatism.^16^^–^^18^ In response to this issue, new IOL designs based in inverted meniscus lenses providing better peripheral optical quality have emerged.^19^^,^^20^

Therefore, because of the importance of peripheral vision for everyday tasks and the various conditions or treatments that can alter peripheral refractive errors, it is necessary to assess the impact of these changes on the mobility performance of tasks that involve peripheral vision. Superimposed peripheral errors have been shown to degrade driving performance.^21^ Because poor vision increases the risk of falling in older people,^22^ and stair negotiation is one of their most challenging daily activity,^23^ it is important to study the effects that reduced peripheral vision can have on stair negotiation. Therefore, the main objective of this study is to evaluate the effects of superimposed peripheral refractive errors on stair negotiation.

## Methods

This experiment was carried out with the approval of the Research Ethics Committee of the University of Murcia and was in accordance with the Declaration of Helsinki. A total of 21 subjects volunteered to participate in the study, 7 women and 14 men. All of them were university students from 18 to 26 years of age (mean age = 22 ± 2 years) and provided written consent of the procedures. Inclusion criteria were never having worn contact lenses or spectacles, never having undergone refractive surgery, and not having any pathology associated with mobility problems. Participants were not screened before the experiment was conducted.

**Figure 1.**
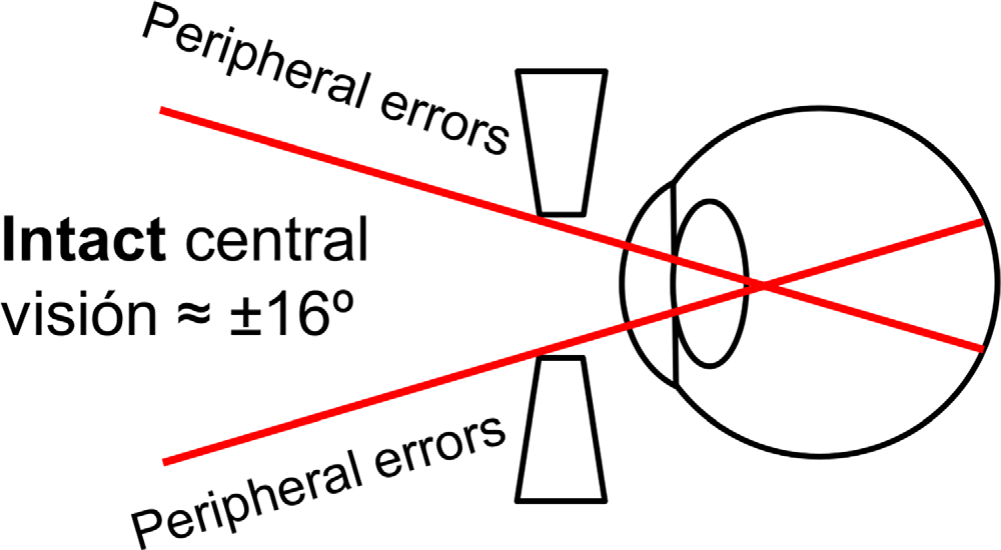
Lens setup. Each lens had a 12 mm diameter central hole, which allowed for intact refraction in the macular area while adding the chosen superimposed refractive errors in the periphery.

To assess the effects of altered peripheral vision on mobility, a step negotiation experiment was designed. Subjects had to walk across an obstacle with steps wearing spectacles that only modified peripheral vision. The spectacles consisted of a standard frame and mounted lenses (1.61 MR8 UV400 HMC; Visionis Distribucion SL, Valencia, Spain) with a specific refractive power and a circular hole of 12 mm diameter in the line of sight of the subjects (Fig. 1). The distance between the center of the holes was approximately 58 mm, in the range of the average interpupillary distance for adults.^24^ The hole in the lens subtended approximately 16 degrees over the retina, thus allowing for unmodified vision in the macular area. The lenses used were: plano lenses, 2 negative defocus lenses (−2 D sphere [SPH] and −4 D SPH), 2 positive defocus lenses (+2 D SPH and +4 D SPH), and 2 cylindrical lenses (+1.75 D CYL and −3.75 D CYL) with axis oriented at 45 degrees. The lenses had a first aspheric surface, thus reducing the peripheral effects of field curvature and astigmatism. However, we also performed measurements with a manual lensmeter (Visionix Inc., France), and found that the power variation in the periphery of the high-power defocus lenses was the appearance of a 0.5 D cylinder with axis zero degrees, of the same sign as the lens power.

**Figure 2.**
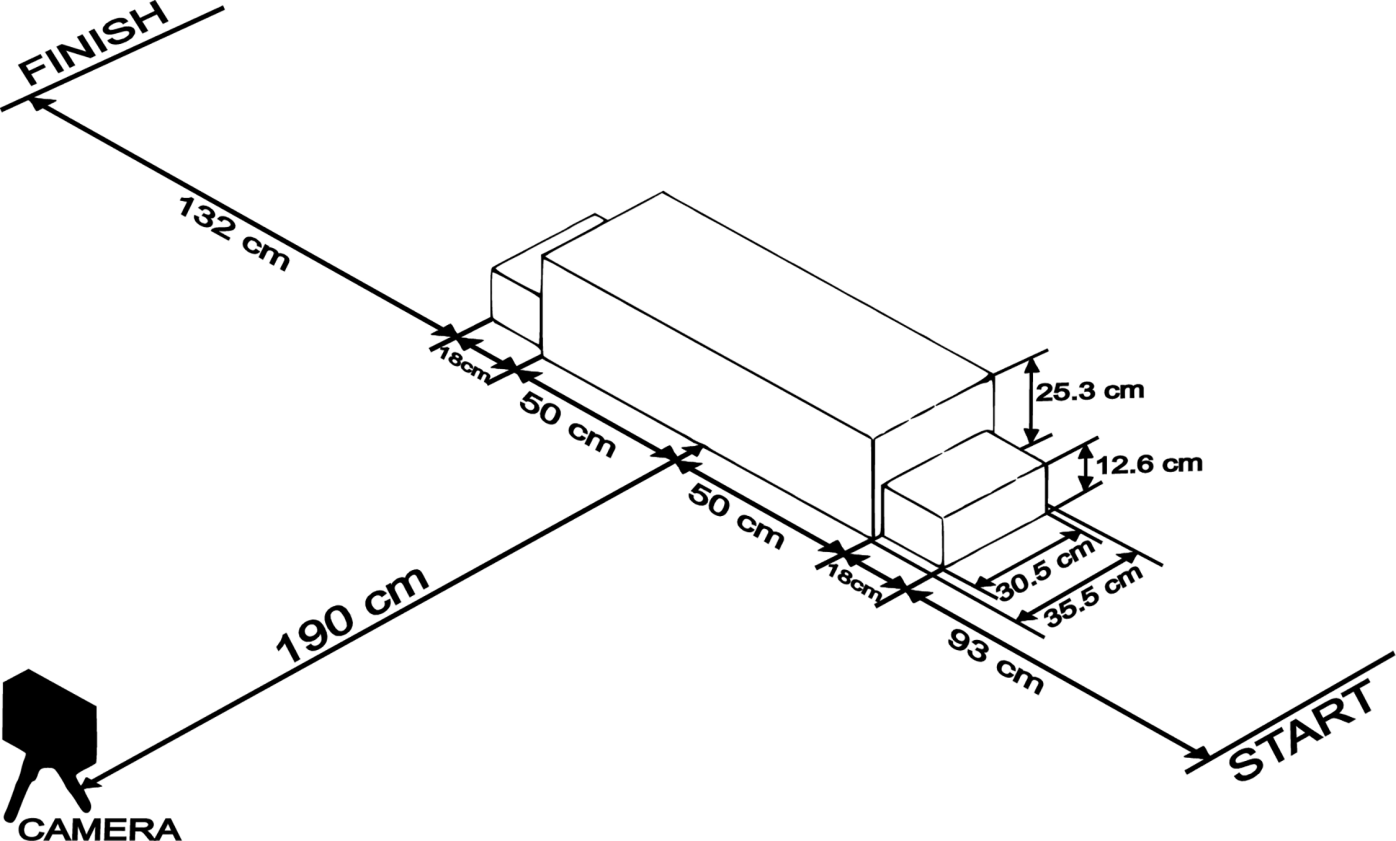
The obstacle setup consisted of an elevated platform with two ascending and descending steps. Subjects walked across it with LED lights attached to their feet, which were used to mark their trajectories. These were recorded with a camera (DMK 37BUX273; The Imaging Source, Bremen, Germany) at 100 fps.

Subjects had to walk from a marked start line, across the obstacle, and arrive to the marked finish line wearing the glasses provided. Details about the setup can be found in Figure 2. LED lights were attached to their shoes, near the heel, to mark the feet trajectory. They were not given any instructions neither on which leg to start the gait, nor on the speed of their walk or the number of steps to take. All the process was recorded at 100 fps using a CMOS camera (DMK 37BUX273; The Imaging Source, Bremen, Germany) with a wide-angle objective.

First, the subjects followed the initial training and went through the path three times without glasses and three more times with the plano lenses. Next, three trials wearing each lens were recorded. The order in which the glasses were worn was from lower to higher refraction power: first, plano lenses, then low-power lenses (+2 D SPH, −2 D SPH, and +1.75 D CYL) in random order, and finally high-power lenses (+4 D SPH, −4 D SPH, and −3.75 D CYL) in random order as well. The order was chosen to minimize the impact of a potential learning effect on the results.

The videos were processed using Kinovea (Charmant and contributors, 2021), a 2-D motion analysis software that has previously been successfully tested for gait analysis,^25^ and Mathematica (Wolfram Research, Inc., Champaign, IL, USA, 2022). Several parameters to characterize the trajectories were obtained:

• Total crossing time: The time from the moment the first foot makes stable contact with the first step until the last foot leaves the last step.
• Total ascent (descent) time: The time between the first foot leaving the ground (platform) to begin ascent (descent) and the two feet reaching the platform (floor).
• Number of steps on the obstacle.
• Ascent and descent speed: Average speed of the trajectory of the foot from the ground to the elevated platform, or vice versa.
• Foot-step placement parameters, such as foot clearance.

**Figure 3.**
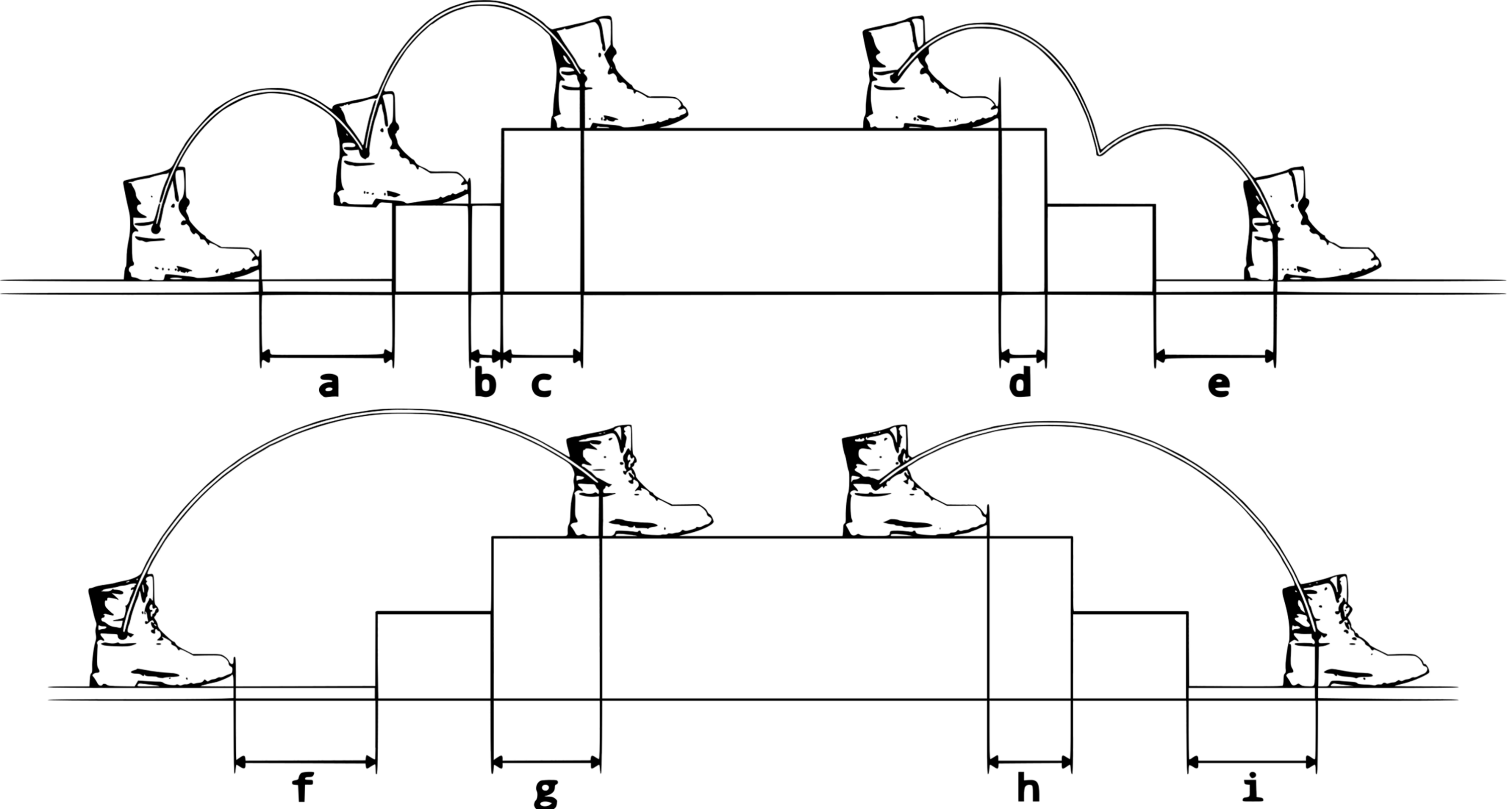
Foot positions measured for the leading foot (*upper figure*) and the following foot (*figure below*). Positions (**a,** **d**) are the starting positions for ascent and descent, respectively, of the leading foot, and (**f,** **h**) are those corresponding to the following foot. (**c,** **e**) These are the landing positions of the leading foot at ascent and descent, and (**g,** **i**) are those corresponding to the following foot. (**b**) This is the intermediate position of the leading foot at ascent.

The foot that initiates ascent and descent will be called the leading foot, and the foot after will be called the following foot. Foot clearance was measured as the minimum Euclidean distance from the LED light to the kerb,^26^ just for the leading foot. Foot placement before and after ascent and descent, for example, starting and landing positions, for the leading and the following foot were measured as well (Fig. 3).

Finally, a repeated measures ANOVA with Bonferroni correction was applied. A sphericity test was performed to check the normal distribution of the residuals,^27^ and if the conditions were not met, a nonparametric Friedman's test was used. All the statistical tests were performed with Jamovi (The Jamovi Project, Sydney, Australia, 2022). As the interest lay in observing the changes in step negotiation between plano lenses and the different superimposed refractive errors, statistical comparisons were made between the types of superimposed peripheral refractive error and the plano lens. Differences were considered statistically significant if *P* < 0.05.

### Peripheral Refraction Measurement

Peripheral refraction was measured using the Voptica Peripheral Refractor (Voptica Peripheral Refractor, VPR, Voptica SL, Murcia, Spain) on 16 of the subjects. The device uses a scanning Hartmann-Shack wavefront sensor to measure refraction, as detailed by Jaeken et al.^28^ For this study, only spherical equivalent (SE) measurements were used.

Measurements were taken at the upper half of the retina (which processes the lower visual field information), at 30 degrees, 15 degrees, and 0 degrees of height, and across a horizontal range of ±30 degrees. The subjects were instructed to fixate their gaze on three different distant points corresponding to the desired elevations to be measured. Three measurements were acquired and averaged for each retinal elevation scenario. Relative peripheral refraction (RPR) was determined by subtracting the central refraction (SE) from the other measured values. To obtain a representative RPR value for each subject at approximately 30 degrees eccentricity, the average refraction from 25 degrees to 30 degrees was measured in both the nasal and temporal retina at an angular height of 0 degrees. For an angular height of 30 degrees, the average refraction was calculated from −4 degrees to +4 degrees. This eccentricity was selected to ensure that peripheral refractive errors superimposed by the spectacles combined effectively, whereas the lenses' aperture did not interfere.

## Results

A full description of the parameters measured, as well as their statistical significance, can be found in Table 1 and Table 2. Statistically significant differences in performance emerged with the addition of all types of peripheral refractive errors.

**Table 1.** Mean ± SD Values of Global and Leading Foot Parameters

	Plano	−2 D SPH	−4 D SPH	+2 D SPH	+4 D SPH	+1.75 D CYL	−3.75 D CYL
Crossing time, s	2.9 ± 0.4	3.1 ± 0.6	3.5 ± 0.7***	3.1 ± 0.5*	3.8 ± 0.8***	3.2 ± 0.5**	3.3 ± 0.5***
Ascent time, s	2.2 ± 0.2	2.2 ± 0.2	2.3 ± 0.3*	2.3 ± 0.2*	2.5 ± 0.3***	2.3 ± 0.2*	2.3 ± 0.3**
Descent time, s	1.9 ± 0.2	1.9 ± 0.2	2.0 ± 0.2	2.0 ± 0.2	2.1 ± 0.2	2.0 ± 0.2	2.0 ± 0.2
Number of steps	4.1 ± 0.3	4.4 ± 0.5*	4.4 ± 0.5**	4.3 ± 0.4	4.6 ± 0.6***	4.3 ± 0.5*	4.6 ± 0.4*
SP ascent, cm	53 ± 17	53 ± 18	42 ± 15**	54 ± 20	50 ± 19	53 ± 19	50 ± 19
Intermediate position ascent, cm	1.8 ± 2.9	1.2 ± 1.9	0.9 ± 1.8	4.6 ± 4.2***	4.0 ± 4.5*	3.7 ± 4.3*	2.4 ± 3.0
SP descent, cm	47 ± 10	40 ± 11*	37 ± 12*	46 ± 10	36 ± 18	43 ± 12	42 ± 10
LP ascent, cm	74 ± 8	69 ± 12	69 ± 11*	67 ± 12**	64 ± 11***	68 ± 11**	68 ± 11**
LP descent, cm	60 ± 7	60 ± 8	57 ± 7	60 ± 10	56 ± 9*	58 ± 7	58 ± 8*
Speed ascent, cm/s	90 ± 13	86 ± 15*	77 ± 14***	84 ± 15**	74 ± 15***	84 ± 13**	79 ± 15**
Speed descent, cm/s	89 ± 10	86 ± 14	80 ± 15*	84 ± 11**	74 ± 15***	82 ± 13*	80 ± 11***
FC first step, cm	21 ± 6	21 ± 7	21 ± 7	22 ± 5	22 ± 6	22 ± 7	22 ± 7
FC second step, cm	31 ± 10	30 ± 11	28 ± 10*	34 ± 10*	33 ± 11	33 ± 11*	32 ± 11
FC third step, cm	18 ± 7	18 ± 6	18 ± 6	16 ± 5	15 ± 6	17 ± 6	17 ± 5
FC fourth step, cm	25 ± 6	24 ± 7	22 ± 6	24 ± 6	24 ± 6	24 ± 6	23 ± 6

Significant *P* values from comparison with the plano lenses are included. Statistical significance is represented in the table in the following way: * if *P* < 0.05, ** if *P* < 0.01, and *** if *P* < 0.001.

FC, foot clearance; LP, landing position; SP, starting position.

**Table 2.** Mean ± SD Values of Parameters for the Following Foot

	Plano	−2 D SPH	−4 D SPH	+2 D SPH	+4 D SPH	+1.75 D CYL	−3.75 D CYL
SP ascent, cm	16 ± 8	14 ± 9	8 ± 7***	17 ± 7	16 ± 8	16 ± 8	15 ± 7
SP descent, cm	1.2 ± 2.5	−0.7 ± 2.8**	0.2 ± 4.4	2.5 ± 3.7	4.0 ± 4.8	2.1 ± 3.7	1.6 ± 3.4
LP ascent, cm	27 ± 8	25 ± 10	24 ± 9	23 ± 10**	21 ± 9***	23 ± 9**	23 ± 10*
LP descent, cm	25 ± 5	24 ± 6	23 ± 6	24 ± 6	23 ± 7	24 ± 5	23 ± 5
Speed ascent, cm/s	148 ± 13	147 ± 15	148 ± 13	142 ± 13*	140 ± 15	145 ± 11	143 ± 13
Speed descent, cm/s	120 ± 14	121 ± 14	118 ± 15	123 ± 14	118 ± 14	116 ± 14	117 ± 11

Significant *P* values from comparison with the plano lenses are included. Statistical significance is represented in the table in the following way: * if *P* < 0.05, ** if *P* < 0.01, and *** if *P* < 0.001.

FC, foot clearance; LP, landing position; SP, starting position.

For the lenses with spherical defocus, the total crossing time increased with respect to the plano lenses as the power of the superimposed error increased: +6.2%, +7.6%, +19.2%, and +29.6% for the −2 D, +2 D, −4 D, and +4 D lenses, respectively (*P* < 0.05 in all the cases, except the −2 D lens). Ascent and descent times followed the same trend, with ascent being more affected by superimposed peripheral errors: +2.3%, +4.7%, +8.4%, and +15.8% for the −2 D, +2 D, −4 D, and +4 D lenses, respectively (*P* < 0.05 for all cases, except the −2 D lens). The number of steps taken on the elevated platform also increased slightly with power, with some subjects taking up to two more steps.

**Figure 4.**
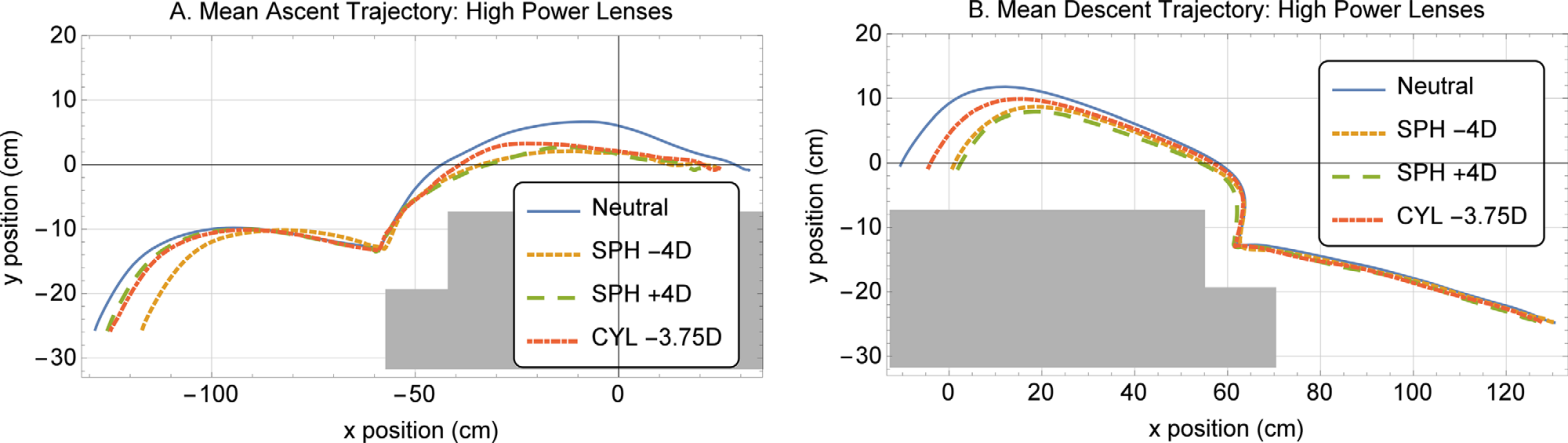
Mean leading foot ascent (*left*) and descent (*right*) trajectories of all subjects with the high-power lenses. Vertical position is on the y-axis, whereas horizontal position is on the x-axis. Steps are represented by a *gray shadow*. *Blue* represents performance with plano lenses, *orange* with negative defocus, *green* with positive defocus, and *red* with the cylindrical ones.

When analyzing mean ascent and descent trajectories, a similar behavior can be observed for the trajectories with all the types of refractive error (even though cylindrical lenses exhibited a less pronounced change in performance): starting and landing positions, as well as maximum foot elevation were generally reduced with respect to the plano lens (Fig. 4). Foot clearances slightly increased with the superimposed peripheral refractive error at ascent and decreased at descent. For ascent, the starting position was especially reduced in the case of −4 D lenses (−20.8% of reduction in distance, *P* = 0.005). During descent, both −4 D and +4 D defocus lenses behaved similarly, being the change with respect to the plano lenses significant for the negative lenses (*P* = 0.02). The position of the foot on the intermediate step was affected by the sign of the defocus: whereas the distance to the bottom of the step increased for high-power positive defocus lenses (+122.2%, *P* = 0.02) it decreased for high-power negative defocus lenses (−50%, *P* = 0.15). Performance differences were more subtle with low power lenses.

For the leading foot, the ascent to the elevated platform (second step) showed the greatest reduction in peak speed. The mean speed for both ascent and descent trajectories were also reduced by superimposing the peripheral errors (see Table 1).

**Figure 5.**
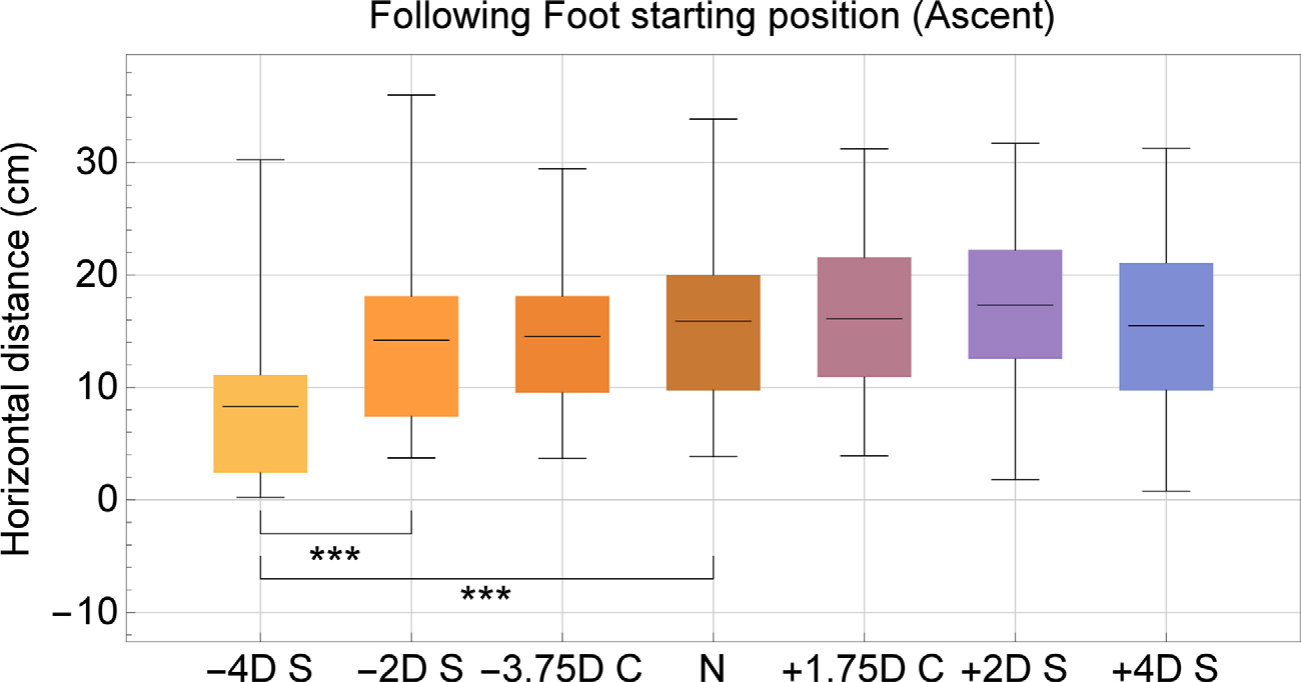
Box and whiskers representation of following foot starting positions at ascent. Distance to the step is represented on the vertical axis, whereas the type of lens is on the horizontal axis. Lenses are ordered by spherical equivalent. Box ranging from the 0.25 quartile to the 0.75 quartile, whiskers extend across the entire dataset, the mean is represented by a *black line*. Statistically significant differences between pairs of lenses are represented in the following way: * if *P* < 0.05, ** if *P* < 0.01, and *** if *P* < 0.001.

The parameters associated with the following foot were less affected by the superimposed peripheral errors (see Table 2), as few statistically significant differences were found. Still, some differences in the placement of the following foot emerged depending on the type of superimposed peripheral error (Fig. 5). Ascent and descent starting distances decreased (compared to the plano lenses) by −52.2% and −83.3% for −4 D of defocus, respectively, whereas for +4 D of defocus, the differences in starting distance decreased −2.5% at ascent and increased +233.3% at descent. That is, on average, the following foot was placed closer to the steps with negative lenses and further away with positive lenses.

### Peripheral Refraction

**Figure 6.**
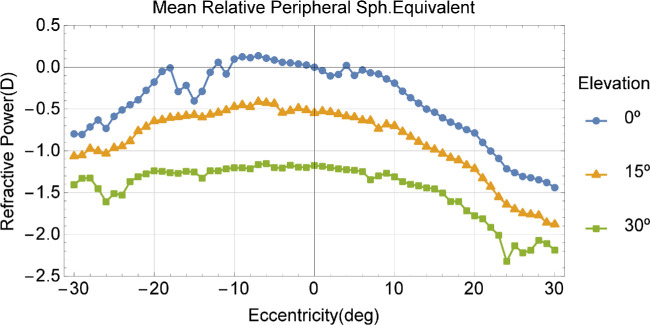
Mean values of RPR for all subjects measured. In *blue*, are the values corresponding to the central meridian, in *orange* are those at 15 degrees of angular height, and in *green* are those corresponding to 30 degrees of angular height. Positive values of eccentricity represent the temporal retina.

Mean central SE was −0.68 ± 0.22 D (range from −0.39 D to −1.15 D) for the right eye and −0.48 ± 0.34 D (range from 0.025 D to −1.14 D) for the left eye. Figure 6 shows the mean relative to the central peripheral refraction of both eyes for all participants. Relative peripheral refraction increased toward more myopic values with increased eccentricity.

**Figure 7.**
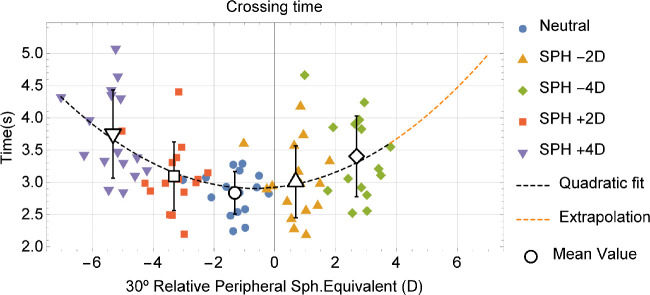
Plots of crossing time (vertical axis) as a function of RPR at 30 degrees eccentricity. The *dashed line* corresponds to the best quadratic fit to the data, this line being *orange* for the extrapolation to unmeasured values of RPR. Different colors correspond to the different lenses worn by the subjects. In *black* and *white* are the mean values and standard deviation of the measurements.

On average, the 30 degrees relative peripheral SE was −1.32 ± 0.70 D. A quadratic fit between some of the parameters measured and the 30 degrees RPR was performed. Figure 7 shows the crossing time versus the peripheral refraction at 30 degrees and a quadratic fit (dashed line). It can be seen how, as the total RPR increases, crossing time increases as well (the best quadratic fit function was crossing time (s) = 2.92 + 0.0476x + 0.0352x²; *R*² = 0.232: *P* < 0.001 for parameter x²).

## Discussion

Overall, we demonstrated here that peripheral defocus affects a daily mobility activity, the negotiation of steps and kerbs. The different types of refractive error (positive defocus, negative defocus, and astigmatism) had common effects on the negotiation of the obstacle: subjects tended to increase the number of steps, slowed down movements, and moved closer to the steps before ascending or descending, as well as reduced foot elevation. However, the magnitude of the changes induced was different depending on the type of lens. Positive high-power lenses had the bigger impact on performance. For cylindrical lenses, there seemed to be a saturation effect with power, as most of the significant differences were observed when compared to plano lenses and not between low and high cylindrical powers. It is known that peripheral detection (high and low contrast) is affected by defocus.^29^ Hence, by superimposing peripheral errors all these features are intensified, and can degrade performance in tasks that rely on peripheral vision. The obtained results showed changes in the gait parameters for the different superimposed refractive errors, and these changes were greater as the peripheral optical quality worsened. The lens that deviated the most from the plano lens was +4 D SPH, followed by −4 D SPH and −3.75 D CYL.

The importance of lower visual field information on gait^30^^,^^31^ and when negotiating steps^10^^,^^32^^,^^33^ has been previously assessed, and it is known that the restriction of lower visual field information causes a cautious locomotor behavior. At this work, by degrading lower visual field information with additional peripheral refractive errors, subjects’ movements became slower and more cautious, largely changing leading foot starting positions and taking more steps, either because of misinterpretation of the scene due to the poor quality of peripheral vision, or as a mechanism of cautious adaptation to the lack of important visual information. Especially on the first step of the descent, the subjects often descended cautiously, touching the edge of the step with their heel to perceive its position and shape (which could also be an explanation for the minor differences on foot clearance). On the other hand, small stumbles or miscalculations of foot position sometimes occurred on the upward steps.

The differences in performance between the positive and negative defocus lenses may be explained by peripheral intrinsic refraction. Given that subjects were approximately −1.3 D myopic on the 30 degrees periphery, as expected from emmetropes,^12^ when wearing the spectacles with positive defocus they were even more myopic on the peripheral retina (approximately −3.3 D and −5.3 D with the +2 D and +4 D lenses, respectively). But when wearing negative defocus spectacles (−2 D and −4 D lenses), the intrinsic myopic defocus at the peripheral retina would be compensated (or even over-compensated). So, it is indeed expected that the overall performance was more affected when wearing the high-power positive lenses (see Fig. 7).

There also seems to be some adaptation to the intrinsic peripheral refraction of each subject,^34^ as subjects with very different peripheral RPR show similar performances, for example, subjects with a higher intrinsic peripheral refractive error do not always perform worse, as shown in Figure 7: when wearing the plano lenses, subjects corresponding to two extreme values of RPR display similar values of the parameter, and it is only when peripheral errors are superimposed, widely increasing peripheral refraction, that the performance is worsened.

On the other hand, the plus/minus lenses generate an opposite magnification effect that can affect the gait. Elliot and Chapman discussed the effect of defocus on gait and concluded that changes in parameters like foot clearance and foot positions are mainly driven by magnification.^35^ In this study, in the case of both ascent and descent, wearing negative spherical lenses made the subjects get closer to the step before beginning the movement. Negative lenses were also the only ones that showed a tendency in reducing the intermediate position of the leading foot at ascent, as well as to reduce the starting positions for the following foot (see Fig. 5). Overall, it suggested that superimposed negative defocus on the periphery induced the need of getting even closer before making any steps, compared to other refractive errors studied here.

Given the actual effects of the superimposed peripheral refractive errors on gait and step negotiation, these results may be relevant in case the peripheral refraction is modified. IOLs implanted in cataract surgery degrade peripheral image quality^36^: they cause greater off-axis astigmatism^17^ and myopic shift in the periphery,^18^ as well as worse hazard detection,^37^ which, according to our results, could lead to difficulties in negotiating stairs. New IOL designs have recently emerged and proved to provide better peripheral quality than regular IOL designs.^19^^,^^20^ Moreover, wearing multifocal glasses increases the risk of falls at stair negotiation in older people,^22^ and these spectacles can increase variability in foot clearance over steps.^38^ However, no significant differences in performance during stair negotiation have been found so far between multifocal and monofocal IOL users,^39^ nor a decrease in mobility parameters.^40^ On the other hand, optical treatments for myopia control consist of modifying the peripheral refraction to introduce myopic defocus, with spectacles, soft contact lenses,^41^ or orthokeratology,^42^ which could potentially also cause mobility issues.

Because the elderly seem to rely more on visual information for guidance when climbing stairs,^43^ these events, which have occurred here in young healthy subjects, could be exacerbated in older subjects, potentially causing falls. The risk of falling is also higher at descent, as it is necessary to rely more on the peripheral lower visual field, and the heel clearance is usually very small.^44^ Moreover, older adults seem to have more variability on foot clearance at descent than young ones, as well as a lack of precautionary increases on foot clearance under reduced lighting.^45^

Some limitations of the current study should be noticed. The small differences in foot clearance could be caused by the placement of just one single marker, so placing one more marker at the tip of the shoe would be positive. Additionally, it might be possible that the design of the glasses could cause uncommon head movements instead of the usual eye movements. Spectacles were the same for all subjects, so the values of mean interpupillary distance were used to design them. However, the spectacles were adapted to each subject via the pad arms to obtain vertical alignment. Another spectacle-like limitation comes from the intrinsic design of the central hole. It induces a refractive jump in the subject's visual perception, which may result in a narrow ring of visual disturbance. This disturbance can arise from both the prismatic effect and diffraction at the edge of the aperture. Finally, the experiment would also benefit from the use of an eye tracker to obtain more information about eye movements. Further research on this topic could focus on exploring the impact that adaptation to the lenses has on performance.

In conclusion, we used a simple but effective experiment to test the effects of peripheral refractive errors on a functional task, such as step negotiation. All the superimposed errors influenced the performance, causing a cautious behavior of the subjects. The differences in performance according to the type of lens used can be explained by the effects of magnification and the intrinsic peripheral refraction of the subjects. These results may be relevant in cases where the peripheral refraction is modified, as in the case of IOL implants, multifocal spectacles, or myopia treatment lenses.
